# A novel, multi-level approach to assess allograft incorporation in revision total hip arthroplasty

**DOI:** 10.1038/s41598-020-72257-3

**Published:** 2020-09-16

**Authors:** Tim Rolvien, Christian Friesecke, Sebastian Butscheidt, Thorsten Gehrke, Michael Hahn, Klaus Püschel

**Affiliations:** 1 Department of Orthopedics, University Medical Center Hamburg-Eppendorf, Martinistraße 52, 20246 Hamburg, Germany; 2 Department of Orthopedics, Tabea Hospital, Kösterbergstr. 32, 22587 Hamburg, Germany; 3 Helios Endo Clinic Hamburg, Holstenstraße 2, 22767 Hamburg, Germany; 4 Department of Osteology and Biomechanics, University Medical Center Hamburg-Eppendorf, Lottestr. 59, 22529 Hamburg, Germany; 5 Department of Forensic Medicine, University Medical Center Hamburg-Eppendorf, Butenfeld 34, 22529 Hamburg, Germany

**Keywords:** Bone, Osteoarthritis, Tissues, Trauma

## Abstract

The successful use of allografts in reconstructive orthopedic surgery, including revision total hip arthroplasty (THA), has been outlined repeatedly. Nonetheless, as previous studies were primarily based on clinical follow-ups, we aimed to create an algorithm that accurately determines the extent of allograft incorporation in the acetabulum and femur using a suite of high-resolution imaging techniques. This study is based on a large patient database including > 4,500 patient data with previous revision THA and simultaneous use of allografts. While the database was continuously matched with the deceased individuals at the local forensic medicine department, complete hips were retrieved in case of a positive match. A positive match was achieved for n = 46 hips at a mean follow-up of 11.8 ± 5.1 years. Comprehensive imaging included contact radiography, high-resolution computed tomography (HR-pQCT), undecalcified histology of ground sections and quantitative backscattered electron imaging (qBEI). We here define a histomorphometric toolkit of parameters to precisely characterize the incorporation of structural (bulk) and morselized (chip) allografts in the acetabulum (n = 38) and femur (n = 8), including the defect area and interface length, microstructural and cellular bone turnover parameters as well as overlap and fibrosis thickness. This collection of samples, through its unique study design and precise definition of incorporation parameters, will provide the scientific community with a valuable source for further in-depth investigation of allograft incorporation and, beyond that, the regenerative potential of this osteoconductive scaffold.

## Introduction

Total hip arthroplasty (THA) represents a commonly performed surgical procedure to restore the joint function of the hip in patients with osteoarthritis (OA) and other joint diseases^1^. Only in Germany, THA is carried out over 250,000 times annually^2,3^. While overall excellent results regarding pain reduction and mobility are achieved, one of the major long-term complications is aseptic loosening of the components of the joint prosthesis (i.e., the acetabular cup and the femoral stem)^4^. Aseptic loosening is usually associated with bone loss around the prosthesis. During revision surgery, the removal of the implants can be accompanied by additional bone loss, which can be a major challenge for the fixation of the new prosthesis.

Allografts are commonly used to restore the host bone stock and to provide sufficient stability for the new prosthesis. Different techniques and types such as morselized (“chips”) and structural (“bulk”) bone allografts can be used depending on the defect size and configuration^5,6^. Although good to excellent short- to long-term clinical and radiological outcomes and survival rates of acetabular and femoral revisions using allografts have been documented in the literature^7–14^, the underlying factors for the success of allograft incorporation are not completely understood. It is likely that the defect size, the surgical technique, the type of allograft, the use of bone cement and host-related factors influence graft incorporation^15^.

In the current article, we present a unique approach to histologically assess the total incorporation potential of allografts used in revision THA. We have managed to include complete hip explants of 46 individuals (83% acetabulum, 17% femur) to precisely characterize the host bone–allograft bone interface. This recruitment procedure with post-mortem explantation allowed us to depict the complete interface region. Thus, we are able to assess different factors that may contribute to allograft incorporation and to compare the histological incorporation of structural allografts and allograft chips.

## Methods

### Recruitment procedure

We included all patients who underwent cemented revision THA (acetabulum, femur) in combination with the use of allografts (chips, structural) at the ENDO-Klinik, Hamburg, Germany, one of the largest European centers for joint arthroplasty, between 1987 and 2009. These patients were included in a central database containing of > 4,500 patient data. From 2009 until today, this database has been continuously matched with the deceased individuals who have been examined by the university forensic medicine. In the case of a positive match and after informed consents and approval of the relatives, the hip (pelvis and femur) was explanted and transferred to our research facilities for further analyses. Exclusion criteria were documented loosening of the prosthetic components as well as evidence of a systemic disorder affecting bone turnover (e.g., hyperparathyroidism or an inflammatory or renal disorder) and local changes within the pelvis (e.g., malignant tumor and Paget disease). Furthermore, death was unrelated to the surgical procedure and included primarily traumatic causes or vascular events (e.g., acute stroke or myocardial infarction). While clinical data including surgical reports and perioperative radiographs were available for all patients, we were able to collect 46 hips of 42 individuals. In four cases, allografts had been used in different surgeries and at different locations. The detailed results of the initial study in 13 structural allografts is published elsewhere^16^. This study was approved by the ethics committee of the “Ärztekammer Hamburg, Germany” (WF-005/09) and complied with the Declaration of Helsinki.

### Surgical technique

**Figure 1:**
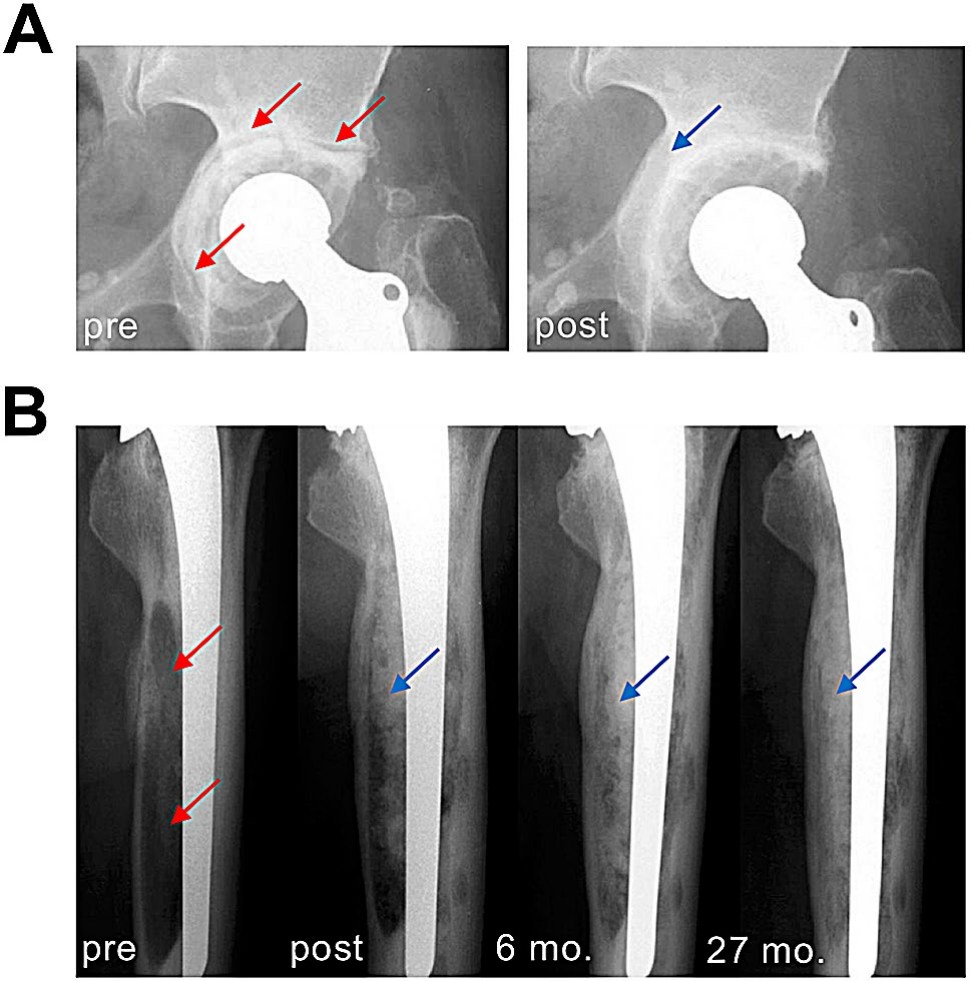
Radiographic appearance of aseptic loosening and surgical reconstruction with allografts. **(A)** Left: Clinical radiograph [left hip, anteroposterior (AP)] showing a cemented cup with circumferential signs of loosening (red arrows), clear central migration with protrusion. Right: Successful reconstruction of the medial wall with allograft chips. The polyethylene cup is sufficiently lateralized (blue arrow). **(B)** Radiographic course (left femur, AP) in a patient with extensive bullous lysis around the femoral stem (red arrows) after cemented THA and increasing bony consolidation after revision THA and reconstruction with impacted allograft chips (blue arrows). *Pre* preoperative, *post* postoperative, *mo*. months.

In all cases, revision THA was performed due to aseptic loosening of the acetabular cup or the femoral stem associated with bone loss (Fig. 1). In the acetabulum, the extent of bone loss varied from minimal destruction (Paprosky 1–2) to more extensive (Paprosky 3) defects^17,18^. Paprosky 3 defects required reconstruction with structural allografts, which were usually additionally fixed with screws. The allograft was adapted as exactly as possible to the bony defect situation. The typical indications for such structural grafts were a bone-deficient acetabular superior dome or medial wall. As large anterior and posterior defects usually cannot be adequately reconstructed with allografts, metal supporting shells were used in these cases. Remaining incongruities were additionally filled with cancellous bone chips if needed. The reconstruction of Paprosky 1–2 defects (Fig. 1A), which is technically easier from a surgical point of view, was performed by preparing cancellous bone chips from the femoral head allograft.

While initially fresh frozen allografts had been used, thermal disinfection was applied from 1994 onwards. Thermal disinfection (101–109 °C for 45 min.) was carried out using a Lobator SD-2 system (Telos, Marburg, Germany) with subsequent cryopreservation at − 80 °C. The femoral head was ensured to be free of cartilage, and larger areas of cortical bone were removed. Subsequently, cancellous allograft chips with an edge length of 3–7 mm and irregular shape and size were prepared. This is necessary to achieve an optimal interlocking with each other and with the bone defect. The mixture of cancellous chips resulted in an ideal granulate of different grain sizes, which were impacted into the bone defect according to the impaction grafting technique described by Sloof et al. ^19^. The allograft chips were carefully degreased before implantation of the cemented cup. By using bone cement, the complete contact surface of the prosthesis could be embedded in the reconstructed acetabulum. Of note, cementless fixation techniques usually require at least 60% contact surface with the host bone^20^.

In the femur, large bone defects (ENDO-Klinik classification Grade 2–4^21^ or Paprosky 2–4^22,23^) were reconstructed with cancellous chips and a cemented stem according to the Exeter technique ^24^ (Fig. 1B).

### Specimen preparation & imaging

**Figure 2:**
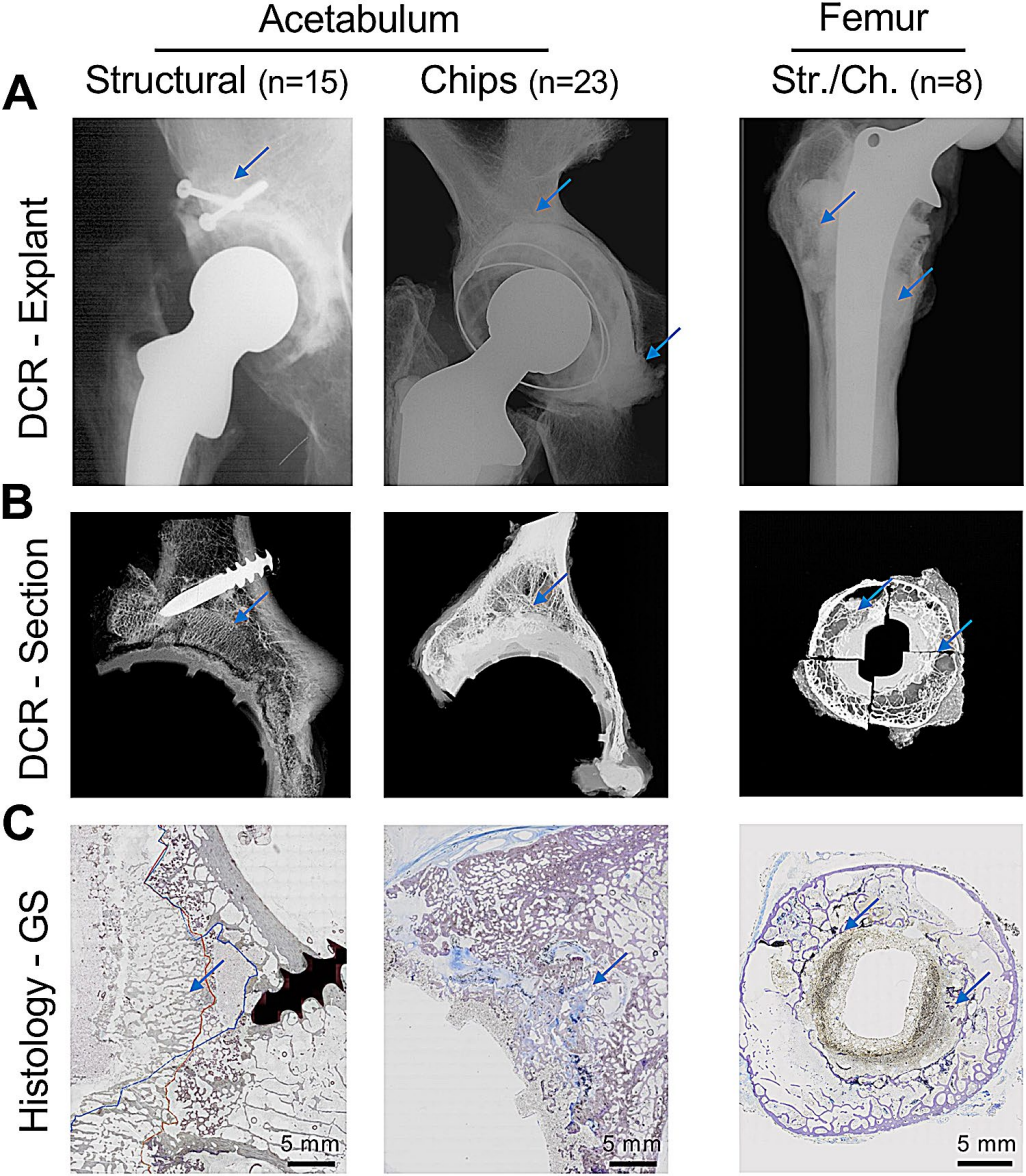
Imaging and processing of the hip explants in 46 cases (acetabulum and femur). **(A)** After explantation of the hip region, digital contact radiography (DCR) of the complete explant was performed first. Blue arrows indicate the allograft localization in accordance with available surgical reports. **(B)** Further processing included DCR of the cut sections, where the allograft region could already be identified (blue arrows). **(C)** Overview images of the ground sections (GS) reveal the bone microstructure of the host bone and the allograft. Blue arrows: allograft localization.

After acquisition of all required clinical data (surgical reports, clinical radiographs), complete removal of the hips and digital contact radiography (DCR, Faxitron X-ray Corp., Wheeling, IL, USA) of the explants was performed (Fig. 2A). The additionally performed three-dimensional (3D) high-resolution computed tomography (HR-pQCT, Scanco Medical, Brüttisellen, Switzerland) of the respective regions of interest (acetabulum or femur) allowed us an exact localization of the transplant. From the identified complete interface region, cut sections were carried out using a diamond band saw (EXAKT Advanced Technologies, Germany). These cut sections were again imaged by contact radiography (Fig. 2B), and ground sections of the complete cut sections were performed according to a previous protocol from our group^25,26^. Briefly, processing of the specimens included fixation in 3.7% formaldehyde, dehydration in an ascending series of ethanol, embedding in a PMMA based plastic medium (Technovit 7200; EXAKT/Kulzer, Germany) and grinding with an EXAKT grinding unit. Ground sections (30 µm) were stained by toluidine blue.

The complete ground sections were scanned using an Olympus BX61 microscope (Olympus, Shinjuko, Tokyo, Japan) equipped with a camera and by merging 500 to 1,000 single images (50 × magnification) to create a large image file covering an overall area of approximately 20 cm^2^ (Fig. 2C). The acquired images were subsequently analyzed by Olympus Stream Motion 2.1 imaging software. Another cut section was subdivided into several parts for undecalcified histology processing (4 µm) and subsequent staining with toluidine blue, von Kossa and trichrome Masson-Goldner.

Next to histology and histomorphometry of the ground and histological sections, our study protocol also involves other micro-morphological techniques to further characterize the host bone–allograft bone interface. Quantitative backscattered electron imaging (qBEI, LEO 435 VP; LEO Electron Microscopy Ltd., Cambridge, England) is operated at 20 kV and 680 pA with a constant working distance. While the beam current is controlled using a Faraday cup, all parameters are kept stable and the grey values are controlled using calibration standards with carbon and aluminum (MAC Consultants Ltd., England). As the generated grey values of each pixel represent the mean calcium content (mean Ca-Wt%), the technique is used to measure the bone mineral density distribution (BMDD) as described previously^27^. Acid etching and subsequent scanning electron microscopy (SEM) will be performed to image the lacuna-canalicular network of the host bone and allograft bone as described^28,29^.

For all individuals, we have additionally prepared histological sections of obtained iliac crest biopsies to assess the overall skeletal status belonging to the respective specimens, which includes bone microstructure and turnover.

### Statistical analysis

Prism software version 7 (GraphPad Software Inc., La Jolla, USA) was used for statistical analysis. Normal distribution of the data was tested with the D'Agostino & Pearson normality test. Datasets were analyzed by one-way ANOVA with Tukey’s post hoc analysis for multiple comparisons in the case of normally distributed data. For non-normally distributed data, the Kruskal–Wallis test with Dunn’s post hoc test for multiple comparisons was performed. All data are reported as individual values (scatter plots) and mean ± standard deviation (SD). Where appropriate, differences between groups were also reported with corresponding 95% confidence intervals (CI). A p-value ≤ 0.05 was considered as statistically significant.

## Results

### Clinical characteristics

**Figure 3:**
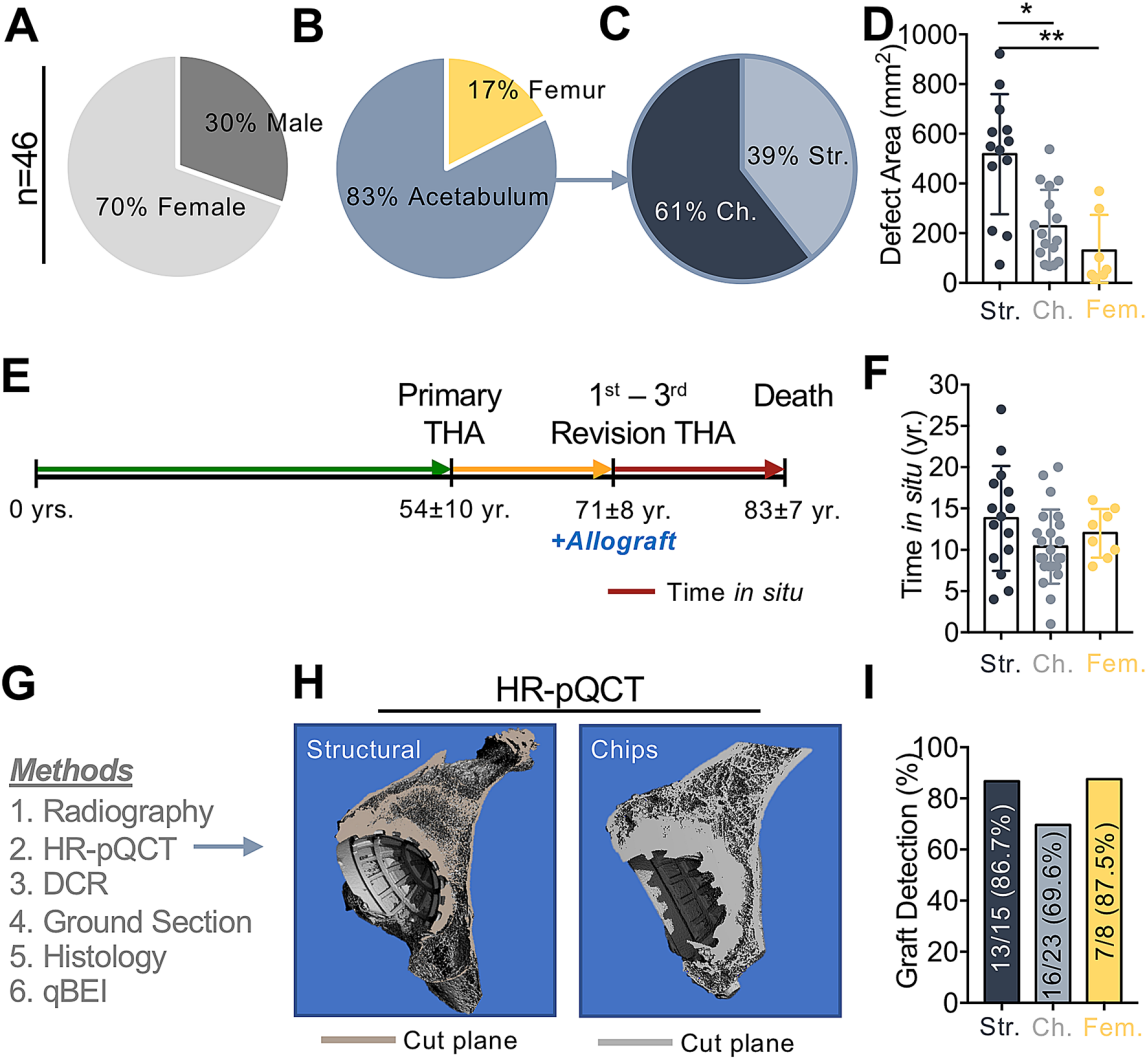
Demographic and disease-specific characteristics. **(A)** Sex distribution. **(B)** Skeletal region in which the allograft was implanted (acetabulum *vs.* femur). **(C)** Distribution of structural allografts (Str.) and allograft chips (Ch.) among the acetabular specimens. **(D)** Quantification of defect sizes in structural (Str.), chip (Ch.) and femoral (Fem.) allografts determined in the ground sections. Kruskal–Wallis test with Dunn correction for multiple comparisons. **(E)** Timeline demonstrating the mean age at primary total hip arthroplasty (THA), allograft use during the revision arthroplasty and analysis of the specimens at the time of death. **(F)** Comparison of the time in situ between structural (Str.), chip (Ch.) and femoral (Fem.) allografts. ANOVA with Tukey correction for multiple comparisons. **(G)** Summary of the conducted methods. HR-pQCT: High-resolution peripheral quantitative computed tomography, DCR: digital contact radiography, qBEI: quantitative backscattered electron imaging. **(H)** Three-dimensional HR-pQCT served for more detailed allograft localization, surface colors indicate virtual cut planes. **(I)** Differences in the success of graft detection (via HR-pQCT and histology). *p < 0.05, **p < 0.001.

Of the 46 specimens included, 32/46 (69.6%) were female and 14/46 (30.4%) were male (Fig. 3A). Thirty-eight of 46 (82.6%) allograft specimens were obtained from the acetabulum, while the remaining 8/46 (17.4%) were allografts in the femur (Fig. 3B). Of the 38 acetabular allografts, 15/38 (39.5%) were structural (bulk) allografts and 23/38 (60.5%) were allograft chips (Fig. 3C). In most cases, polyethylene acetabular revision cups were implanted. Metal supporting shells (e.g., Burch-Schneider rings) were only used in 4 cases (2/23 chips, 2/15 structural). Additional synthetic grafts (hydroxyapatite, tantalum or glass ionomer) were used in 4/23 (17.4%) chips, 2/15 (13.3%) structural and 2/8 (25.0%) femoral allografts. The defect area determined in the ground sections was significantly larger in the specimens with previous use of acetabular structural allografts compared to acetabular chip allografts (p = 0.012) and femoral allografts (p = 0.0007), while no differences between acetabular chip allografts and femoral allografts were observed (Fig. 3D).

The mean age at primary THA was 54.4 ± 10.4 years. While the allografts were used during the 1st to 3rd revision THA at an age of 71.2 ± 8.2 years, the mean age of death was 83.0 ± 7.5 years (Fig. 3E). The resulting time that the allografts remained in the individuals’ body (i.e., the time in situ) varied from 1.2 to 27.1 years (11.8 ± 5.1 years). No differences in the time in situ between structural allografts, allograft chips and femoral allografts could be determined (mean difference structural *vs*. chips 3.4 years (95% CI − 0.6 to 7.4 years) p = 0.11; mean difference structural *vs*. femoral 1.8 years (95% CI − 3.5 to 7.1 years) p = 0.69; mean difference chips *vs*. femoral − 1.6 years (95% CI − 6.5 to 3.3 years) p = 0.11) (Fig. 3F). The individual data of the included 46 cases for structural and chip allografts (both acetabular) as well as femoral allografts is displayed in Supplementary Table 1.

### Graft detection & definition of incorporation parameters

We have applied several methods for optimal graft detection (Fig. 3G). After initial localization of the grafts from the surgical reports and clinical radiographs, allografts were imaged in 3D by HR-pQCT (Fig. 3H). This enabled us to determine cut sections with the largest possible interface for further histomorphometric analyses. Graft detection was achieved in 13/15 (86.7%) of the structural allografts and in 16/23 (69.6%) of the allograft chips in the acetabulum as well as in 7/8 (87.5%) of the allograft chips in the femur (Fig. 3I).

**Figure 4:**
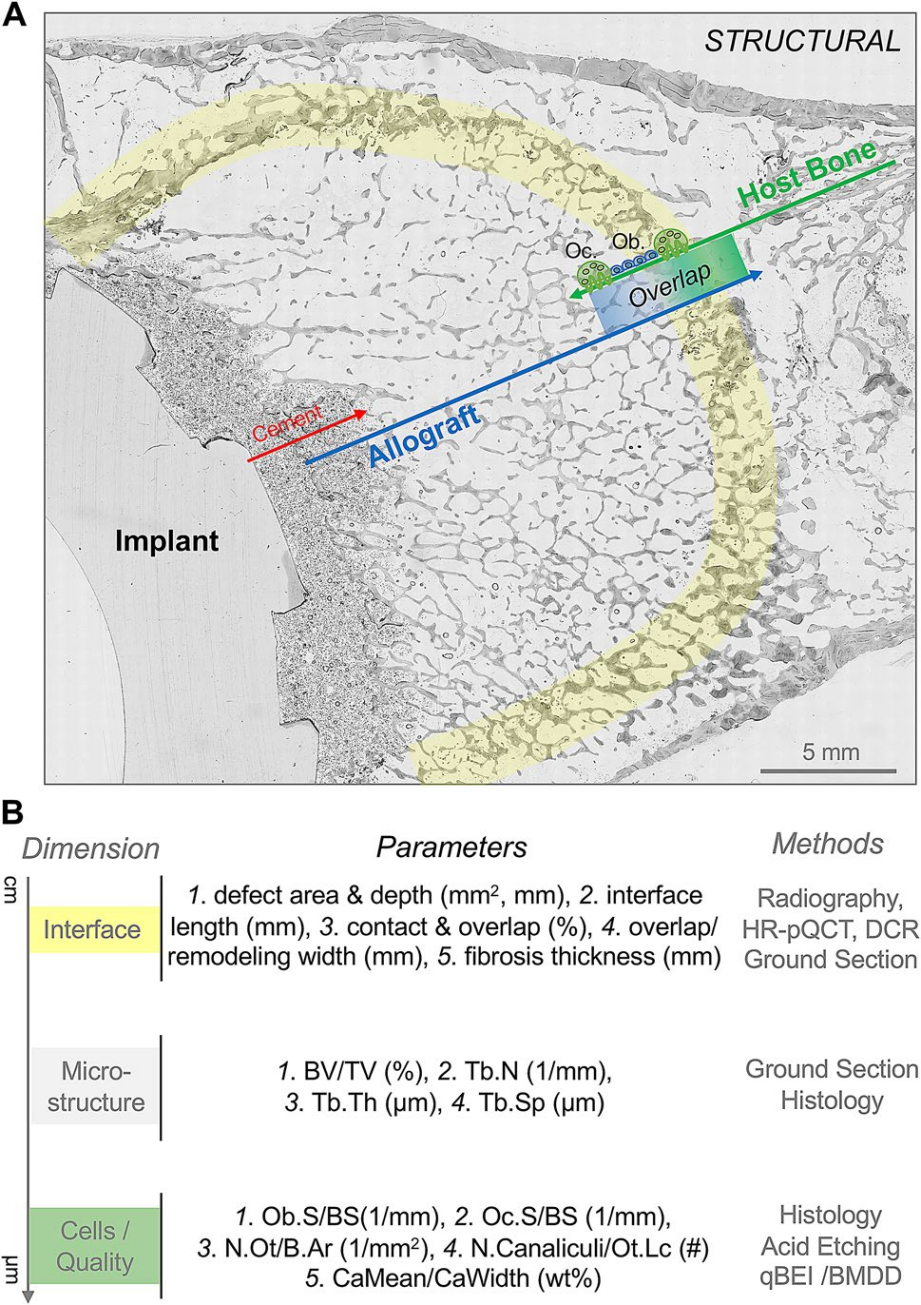
Toolkit of parameters for the detailed analysis of allograft incorporation. **(A)** Overview of a ground section with a large, well-incorporated structural allograft (STRUCTURAL) with a schematic illustration of the incorporation analysis. The direct contact as well as the overlap between the host bone and allograft can be quantified from large high-resolution imaging files. The images also enable to visualize and quantify the cement to allograft bone interface, the implant as well as the microstructure of the different regions. *Ob.* osteoblast, *Oc.* osteoclast. **(B)** Along the different dimensions (mm to µm), several parameters to characterize the interface (length and width measurements), the microstructure and the cellular characteristics are defined. These parameters can be obtained using the respective different methods (right column). *BV/TV* bone volume per tissue volume, *Tb.N* trabecular number, *Tb.Th* trabecular thickness, *Tb.Sp* trabecular separation, *Ob.S/BS* osteoblast surface per bone surface, *Oc.S/BS* osteoclast surface per bone surface, *N.Ot/B.Ar* number of osteocytes per bone area, *N.Canaliculi/Ot.Lc* number of osteocyte canaliculi per osteocyte lacuna, *CaMean* mean mineralization, *CaWidth* mineralization heterogeneity.

A representative ground section overview image of an acetabulum with structural allograft and the definition of the respective histomorphometric incorporation parameters is provided in Fig. 4. From the high-resolution images of the complete ground sections (Fig. 4A), histology and qBEI images, the following quantitative parameters are defined and measured through digital image analysis (Fig. 4B):

1. Interface characterization: Several measurements can be obtained from the ground section images: Defect area & depth (mm^2^, mm), interface length (mm), direct contact of host bone and allograft bone (% of the interface length), overlap or remodeling width (mm) indicating the distance between the most distant avital remnants from the allograft bone body and the maximum ingrowth of the vital host bone penetrating the allograft bone.
2. Microstructure: The structural integrity of the host bone and allograft bone are characterized by the following parameters: Bone volume per tissue volume (BV/TV, %), trabecular number (Tb.N, 1/mm), trabecular thickness (Tb.Th, µm), trabecular separation (Tb.Sp, µm).
3. Cells/bone quality: The interface region, including the allograft and the host bone part, can be furthermore assessed on a cellular and bone quality level by cellular histomorphometry and qBEI. This includes: Osteoblast surface per bone surface (Ob.S/BS, %), osteoclast surface per bone surface (Oc.S/BS, %), number of osteocytes per bone area (N.Ot/B.Ar, 1/mm^2^), number of osteocyte canaliculi per osteocyte lacuna (N.Canaliculi/Ot.Lc, #), BMDD characteristics including the mean mineralization (CaMean, wt%) and mineralization heterogeneity (CaWidth, wt%).

### Interface analysis, cement, fibrosis and graft stability

**Figure 5:**
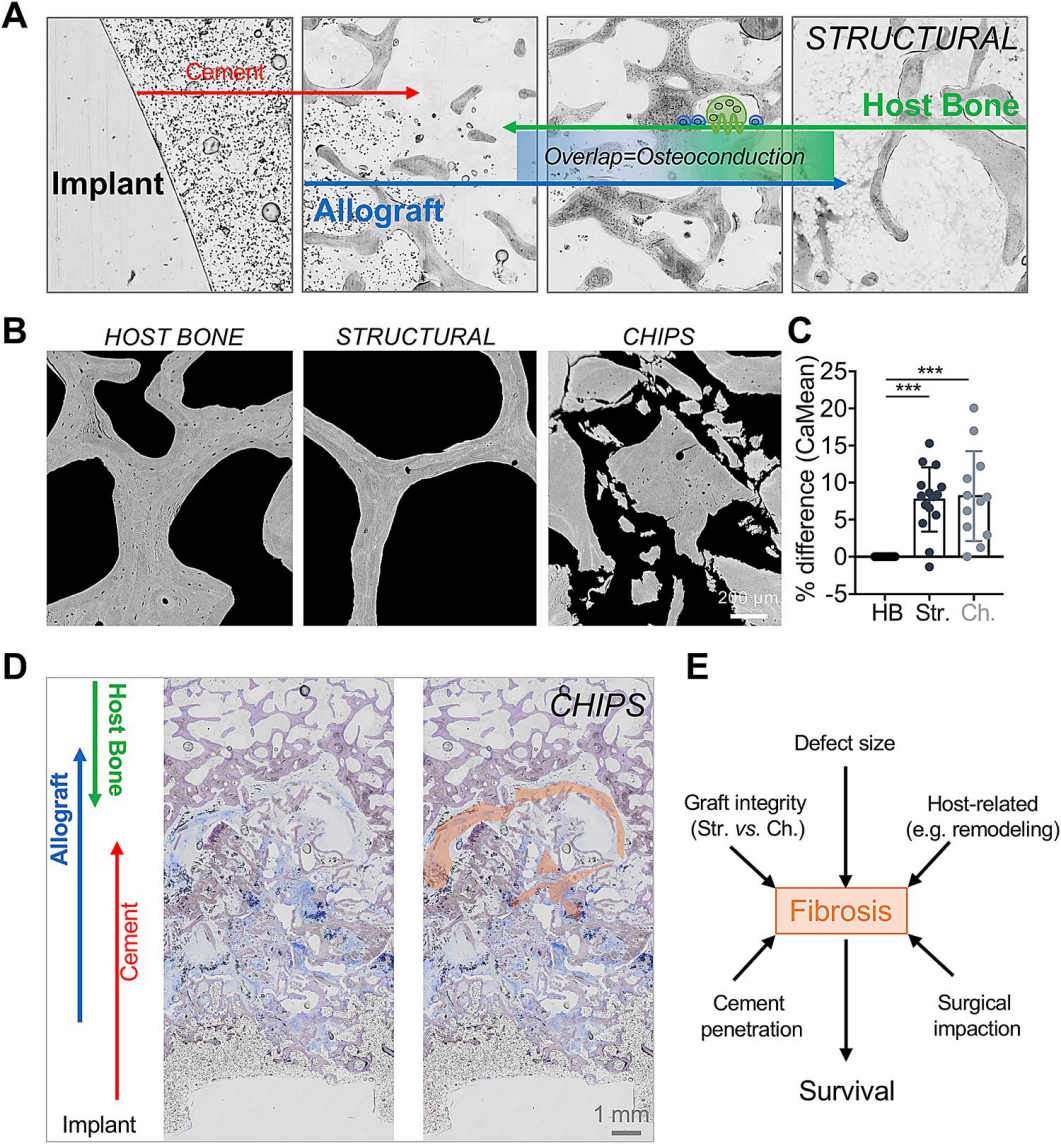
High-resolution imaging enables allograft detection and characterization. **(A)** In higher magnification, the individual areas (implant, cement, fibrosis, allograft, overlap, host bone) are well defined. **(B)** Assessment of bone mineral content by qBEI. Representative images demonstrate the differences in microstructure and mineralization distribution between host bone, structural and allograft chips. **(C)** Quantification of the mean mineralization (CaMean, % change compared to host bone (HB)). Both structural (Str.) and chip (Ch.) allografts show higher CaMean values. ANOVA with Tukey correction for multiple comparisons. ***p < 0.0001. **(D)** Characterization of the host bone–allograft interface in a case with previous implantation of allograft chips (*CHIPS*) (ground section, toluidine blue staining). In this case, large areas of fibrosis are visible, right image: orange markings illustrate the fibrous areas. **(E)** Outline of possible factors influencing the formation of fibrosis and thus the potential stability.

Our study algorithm enables a precise determination of the histological allograft incorporation through digital image analysis of large overview image files of the ground sections. This is achieved by an unmissable appearance of the implant, bone cement, fibrosis as well as dead allograft bone and viable host bone (Fig. 5A). Additional qBEI studies complement the algorithm, since allograft bone has a unique imaging appearance compared to host bone including hypermineralized osteocyte lacunae and significantly higher matrix mineralization of structural and chip allografts compared to the host bone (both p < 0.001) (Fig. 5B, C). In the case of impacted allograft chips, the bone cement can theoretically penetrate deeper into the allograft, while the chips may have a segmented appearance (Fig. 5D). The defined parameters adequately represent the defect size, host-related factors such as bone remodeling capacities, surgical impaction and cement penetration as well as the integrity of the graft, which can all be quantified in relation to the occurrence of fibrosis (Fig. 5E).

## Discussion

It is widely accepted in the orthopedic community that allografts represent a suitable option to address small to large acetabular or femoral bone defects, which represent a common problem in revision procedures (especially revision THA). This notion is primarily based on clinical and radiological evidence with good to excellent outcomes for structural and chip allografts in both the acetabulum^9,19,30–33^ and the femur^14,19,34–36^. On a histological level, previous studies in the acetabulum were limited to small n-numbers and indicated remained integrity of structural allografts with minimal remodeling^37^. While other histological studies have analyzed the incorporation of allograft chips in the acetabulum and the femur, they were all based on small core biopsies. These studies demonstrated new trabecular formation within the grafted area accompanied by fibrous ingrowth at mid- to long-term follow-up^8,38,39^. In the femur, a previous case report indicated that the implanted allografts chips had been largely replaced by viable cortical bone^40^.

We here present a novel, multi-level approach to assess the total incorporation of both structural allografts and allograft chips previously implanted in the acetabulum as well as allograft chips implanted in the femur. Since this approach is based on the combination of clinical data and high-resolution histological analyses in the context of a post-mortem cadaveric study that included complete removal of the respective hips, we are able to assess the complete interface region. To this end, the aim of the present study was to define several incorporation parameters that fully define the complete interface, microstructure and cellular processes. The aim of future studies on these specimens is to compare the allograft incorporation (i.e., the osteoconductive capacity) of structural allografts with allograft chips and of acetabular with femoral allografts. While we have already applied a selection of these parameters in an initial study on structural allografts in which we demonstrated successful incorporation with avital remnants of allograft bone in the center^16^, several clinical questions remain that can be answered with this unique collection of samples.

First, we aim to apply this approach in more detail to the collection of allograft chips (n = 23) as well as to the obtained femoral allografts (n = 8) in order to determine precise similarities and differences regarding the allograft incorporation. For example, although load has been found to have only minor influences on graft incorporation^41^, this study was based on a goat model with only short-term follow-up. It is generally known that mechanical loads have a remarkable impact on skeletal remodeling and overall integrity^42^. Especially the comparison between the acetabular and the femoral specimens could help to answer this question.

Second, as allograft particle size has been found to influence allograft incorporation^43^, the comparison between structural and chip allografts is of major interest. The definition of quantitative and reproducible parameters such as the host bone-allograft bone overlap, the fibrosis thickness as well as cellular parameters of bone turnover (i.e., osteoblasts, osteoclasts) is highly important to detect differences regarding incorporation and osteoconductive capacities. Specifically, it is important to find out whether critical size defects exist, in which structural allografts could be superior to allograft chips in terms of minimal fibrous areas and successful remodeling of the graft. The primary defect area is indeed larger in the specimens with previous use of structural allografts compared to allograft chips, however, the range of defect sizes is sufficient to perform inter-group comparisons. One of the main aims of future studies on the acquired sections is to determine factors that contribute to fibrous ingrowth, which most likely affects graft stability/instability. Furthermore, as there have been conflicting statements regarding the negative effects of cement on allograft incorporation, the cement-allograft bone interface could also be assessed in the present collection of samples.

Third, the time-dependency of both allograft incorporation and potential loosening/failure is not yet completely understood. It has been demonstrated that allograft incorporation may occur early (i.e., in the first weeks) with progressive loosening at long-term follow-up^44^. As this observation was based on a goat model and a maximum follow-up of 4 years, our collection of samples should provide information regarding this issue. Importantly, according to the initial findings of the current study, none of the implants showed a higher degree of loosening or collapse of the graft. Clinically, while low revision rates due to cup loosening have been detected in the midterm around 5 years^31^, in long-term follow-up there was a relevant rate of re-revision and loosening^45,46^.

We are aware that the defined parameters can only provide information regarding cellular and structural incorporation as well as other bone quality aspects but not clinical information such as re-loosening or re-revision rates. THA was not causal for death in any of the cases and only cases without clinical documentation of implant or transplant failure were included, which limits potential investigation into the factors contributing to failure. Nonetheless, it is conceivable that future experimental and biomechanical methods could also be applied on our specimens in order to test the stability of the interface region including the remodeled compared to the non-remodeled allograft as well as to areas that are limited to bone cement without graft material. Other limitations of the present multi-level approach include the fact that allograft bone could not be detected in a number of specimens, although its use had been documented in the corresponding surgical reports. The reasons for this could be either complete graft resorption/remodeling or inaccurate localization of the allograft. Nonetheless, the current algorithm including radiographic studies combined with three-dimensional HR-pQCT and ground sections is most likely the best possible way to achieve the highest possible percentage of allograft detection.

Taken together, this study provides a detailed overview and definition of histological incorporation parameters in a large cohort of patients with allograft use in revision THA. The collection of samples will serve the scientific community as a valuable source for further investigation including in-depth analysis of allograft incorporation in the acetabulum and femur. As the results will be analyzed as a function of different factors such as defect size or the use of structural allografts *vs.* allograft chips, important clinical implications can be derived in the future. This procedure primarily also provides the first sufficient quality control for the successful use of allografts in revision THA.

## Supplementary information

Supplementary Information.

## Data Availability

The data that support the findings of this study are available from the corresponding author upon reasonable request.
