# Supplementary material for: A novel, multi-level approach to assess allograft incorporation in revision total hip arthroplasty
Source: Sci Rep. 2020 Sep 16;10:15226. doi: 10.1038/s41598-020-72257-3 (PMC7494851; doi:10.1038/s41598-020-72257-3)
Supplement: Supplementary file 1 — Supplementary Information. [file 41598_2020_72257_MOESM1_ESM.pdf]

Supplementary Material

A novel, multi-level approach to assess allograft incorporation in revision total hip arthroplasty

Tim Rolvien, Christian Friesecke, Sebastian Butscheidt, Thorsten Gehrke, Michael Hahn and Klaus Püschel

Supplementary Table 1: Individual data of the included 46 cases displayed separately for structural and chip allografts (both acetabular) as well as femoral allografts.

	Number	Bone graft	Age at death (yr.)	Sex	Age at bone graft (yr.)	Time *in situ* (yr.)	Special features	Defect depth (mm)	Defect area (mm²)
Acetabulum – Structural	1	yes	81	f	67	14	Metal shell	6.0	74.1
	2	yes	81	m	66	15	Glassionomer	17.8	615.6
	3	yes	95	f	73	22		16.1	798.7
	4	yes	73	f	66	7		24.6	697.3
	5	yes	65	m	50	15		19.6	493.9
	6	yes	91	f	79	12		18.3	606.9
	7	yes	80	f	61	19		15.4	549.3
	8	yes	74	f	69	5		25.9	922.1
	9	yes	87	f	74	13		19.2	534.9
	10	yes	84	f	67	17		11.2	209.5
	11	yes	88	m	79	9		18.0	570.6
	12	yes	83	f	79	4	Tantalum	14.1	469.9
	13	yes	83	f	73	10	Metal shell	9.9	188.4
	14	no	90	f	63	27		-	-
	15	no	84	f	66	18		-	-
Acetabulum – Chips	16	yes	84	f	73	11	Metal shell	7.4	85.3
	17	yes	93	f	85	8		8.2	122.0
	18	yes	86	f	72	14		12.9	232.6
	19	no	78	m	64	14		-	-
	20	no	89	f	77	12		-	-
	21	yes	99	m	91	8		10.3	259.5
	22	yes	86	f	77	9	Tantalum	4.8	67.4
	23	yes	90	m	80	10	HA	14.7	390.9
	24	yes	62	f	55	7	Metal shell	11.6	197.7
	25	no	75	m	74	1	Tantalum	-	-
	26	yes	85	f	66	19		7.4	158.0
	27	yes	72	f	68	4		14.8	316.0
	28	yes	84	f	73	11		11.6	170.8
	29	no	78	f	66	12		-	-
	30	yes	82	f	76	6	HA	14.5	416.0
	31	no	82	f	62	20		-	-
	32	no	90	f	81	9		-	-
	33	yes	76	m	63	13		9.4	142.1
	34	no	82	f	73	9		-	-
	35	yes	82	f	74	8		14.8	412.0
	36	yes	88	m	71	17		5.1	71.0
	37	yes	86	f	78	8		4.7	72.6
	38	yes	90	f	81	9		17.8	537.5
Femur	39	yes	84	m	75	9	HA	6.3	299.1
	40	yes	81	m	71	10		2.7	53.4
	41	yes	82	m	66	16	Glassionomer	10.2	369.8
	42	yes	77	m	63	14		2.4	32.7
	43	no	94	f	83	11		-	-
	44	yes	86	m	73	13		4.7	102.8
	45	yes	70	f	55	15		1.9	33.6
	46	yes	86	f	78	8		1.8	22.3
